# MiR-221 Promotes Hepatocellular Carcinoma Cells Migration via Targeting PHF2

**DOI:** 10.1155/2019/4371405

**Published:** 2019-05-12

**Authors:** Yi Fu, Mingyan Liu, Fengxia Li, Li Qian, Ping Zhang, Fengwei Lv, Wenting Cheng, Ruixing Hou

**Affiliations:** ^1^Department of Human Anatomy, Histology and Embryology, School of Biology and Basic Medical Sciences, Soochow University, Suzhou 215007, China; ^2^School of Medicine, Yangzhou University, Yangzhou 225001, China; ^3^Institute of Hand Surgery, Ruihua Affiliated Hospital of Soochow University, Suzhou 215007, China

## Abstract

MicroRNAs (MiRNAs), which regulate the gene expression leading to translational inhibition or mRNA degradation, are involved in carcinogenesis and tumor progression. Previous studies have demonstrated that miR-221 was one of the most consistent overexpressed miRNAs in several types of cancer. However, the role of miR-221 in human liver cancer progression is not yet fully elucidated. Levels of miR-221 and plant homeodomain finger 2 (PHF2) expressions in human hepatocellular carcinoma (HCC) tissues and cell lines were detected using western blotting and quantitative real-time PCR (qRT-PCR). Cell migration was studied using the transwell assays. A dual-luciferase reporter system was used to validate the target gene of miR-221. The results indicated that miR-221 promoted HCC cell migration. By performing subsequent systematic bioinformatic analyses, we found PHF2 was the target gene of miR-221 and the direct binding relationship was further validated by dual-luciferase reporter assay. In addition, lower expression of PHF2 promoted HCC cell migration and linked to worse overall survival in HCC patients. Finally, the negative correlation between miR-221 and PHF2 expression levels in HCC specimens was further confirmed. Taken together, our findings implied that miR-221 could be a potential candidate for the therapeutics of HCC metastasis.

## 1. Introduction

Hepatocellular carcinoma (HCC) is the fifth common cancer and third leading cause of cancer-related deaths worldwide, and it is considered to be one of the most common cancers with poor prognosis [1]. Due to the rapid development of sequencing technology, there is a growing comprehension on the molecular mechanisms resulting in HCC carcinogenesis. Previous studies have considerably focused on study of DNA mutations and gene expression changes in HCC [2]. Moreover, RNA mutations and change of mRNA transcription are also correlated with the initiation and progression of HCC.

MicroRNAs (miRNAs) are small noncoding RNAs with approximately 22 nucleotides that could play important regulatory roles in plants and animals by targeting mRNAs for translational suppression [3]. In excess of 2,000 miRNAs have been identified to regulate a variety of protein coding transcription [4]. By modulating gene expression via posttranscriptional mechanisms, miRNAs are now known as vital players in cell cycle, differentiation, apoptosis, and oncogenesis [5]. Furthermore, miRNAs are identified to be correlated with the regulation of epithelial-mesenchymal transition and tumor metastasis by targeting important genes [6, 7]. MiR-221, which is encoded by human chromosome Xp11.3, is often abnormally expressed and associated with the regulation of oncogenes or tumor suppressive genes. Among numerous miRNAs, the upregulation of miR-221 has been recently recognized in numerous types of human cancers [8, 9]. Thus, identification of the function of miR-221 and its targets could make a new access to cancer treatment.

The aim of our study was to investigate miR-221 expression in HCC cells and tissues and to observe the changes in the migration ability of HCC cells following variation of the miR-221 expression. Our data, for the first time, revealed the tumorigenesis role of miR-221 in HCC and identified a target gene plant homeodomain finger 2 (PHF2). PHF2, which maps to human chromosome (Chr) 9q22 [10], is a member of the KDM7 histone demethylase family that contains a plant homeodomain (PHD) in the Jumonji-C and N-terminal domain [11]. Notably, previous studies indicated that PHF2 acts as a cancer suppressor by regulating p53 in colon cancer tissues [12]. However, the role of PHF2 in HCC remains to be investigated. In our study, we demonstrated that miR-221 promoted HCC cells migration via targeting PHF2 and could be a new target for HCC therapeutics. Taken together, our results may provide critical strategy for targeted therapy and prognosis of HCC.

## 2. Materials and Methods

### 2.1. Patients and Specimens

60 patients with hepatocellular carcinoma who underwent resection were collected from Affiliated Hospital of Yangzhou University between 2014 and 2017. The tissue microarray (TMA) consisted of 60 surgical cases produced by the National Engineering Centre for Biochip (Shanghai, China). The patients' clinicopathologic parameters including tumor diameter, sex, age, TNM stage, lymph node metastasis, and depth of invasion. These data were acquired from the Medical Record of the Affiliated Hospital of Yangzhou University. 3-year clinical follow-up data were obtainable for 60 patients from the Yangzhou area. The median follow-up time is 20 months. And the cases of TMA include 2 lost follow-up patients. All the tissues were collected for the present study with patients' informed consent. And the study of human specimens was approved by the Review Board of the Affiliated Hospital of Yangzhou University.

### 2.2. Tissue Samples

36 patients with histologically conformed hepatocellular carcinoma tissues (HCT) were obtained from the first Affiliated Hospital of Yangzhou University. The cancer tissues and adjacent cancerous tissues were collected from patients. All samples were acquired at the time of surgery and were frozen in liquid nitrogen immediately. This investigation was approved by the medical ethics committee of Yangzhou University and informed consent was gained from patients before recruitment.

### 2.3. Immunohistochemistry

Immunohistochemistry was performed with a standard avidin biotinylated–HRP complex (ABC) kit (Zhongshan biotech, Beijing, China) following the ABC method. The slips were incubated with anti-PHF2 antibody (1:1000) (abcam) overnight at 4°C, and diaminobenzidine (DAB; Zhongshan Biotech, Beijing, China) was used to turn out a brown precipitation. The immunoreactivity was evaluated blindly by three pathologists using light microscopy (Olympus BX-51 light microscope), and the image was collected by Camedia Master C-3040 digital camera. The level of PHF2 was ranked as high when ≧5% of tumor cells showed immunopositivity. Biopsies with <5% tumor cells immunostaining were regarded as low.

### 2.4. Cell Lines and Culture

HCC cell lines SMMC-7721, Bel-7402, MHCC97, and HepG2 cells were obtained from the American Type Culture Collection (ATCC). Human normal hepatocyte HL-7702 cells were also obtained from the ATCC. HL-7702, SMMC-7721, and Bel-7402 cells were cultured in RPMI-1640 (GIBCO, US). HepG2 and MHCC97 cells were cultured in DMEM (GIBCO, US). The culture media were in humidified air with 5% CO2 at 37°C, supplemented with 10% fetal bovine serum and 1% streptomycin/penicillin.

### 2.5. Western Blot

After transfection, cells were harvested from the plates. Equivalent proteins were separated by 10% SDS polyacrylamide gel electrophoresis (SDS-PAGE); the proteins were then transferred onto PVDF membranes. After incubated overnight at 4°C with appropriate primary antibodies, the membranes were further probed with a horseradish peroxidase-conjugated secondary antibody (1:2000) for 2h at room temperature. The membrane was detected by enhanced chemiluminescence (ECL) solution and scanned on the chemiluminescence imaging analysis system (Tanon Biotechnology, Shanghai, China). Each western blot was repeated three times.

### 2.6. RNA Extraction and Quantitative Real-Time PCR (qRT-PCR)

After cell transfection, the cellular RNA was extracted from cell lines or tissues using TRIzol reagent (Sigma). Then the RNA transcribed into cDNA by PrimeScript RT master Mix (Takara, Dalian, China) following the corresponding protocols. qRT-PCR was carried out with a SYBR GREEN MIX kit (Promega, Madison, USA) conforming to the manufacturer's instructions. The qRT-PCR detection was carried out using the ABI 7500 FAST Real-Time PCR System. GAPDH was used for loading control. The relative level was calculated using the relative quantification equation (RQ) = 2^−ΔΔCt^.

### 2.7. Wound Healing Assays

SMMC-7721 cells were grown to 80% proportion in 6-well plates wounded by scratching the cell monolayer with a sterilized 200 *μ*l pipette tip. Phase contrast images were collected in the same field at indicated time periods (0, 24, and 48 hours) using the Nikon Digital Microscope with magnification of × 100. Experiments were performed in thrice.

### 2.8. Cell Migration Assays

Cell migration assays were performed by modified two-chamber plates with a pore size of 8 *μ*m. Cells were seeded in serum-free medium in the upper chamber at a density of 1 × 10^5^ cells/well. After 24 h incubation in 37°C, the cells were fixed in methanol and stained with trypan blue. Cells in the upper chamber were carefully removed with a cotton swab and the cells that traversed the membrane were counted under a microscope in five fields. The analysis was performed thrice.

### 2.9. Dual-Luciferase Report Assay

MiRNA-binding regions of PHF2 for miR-221-3p in the 3′-UTR were subcloned into the GP-miRGLO luciferase miRNA vector. SMMC-7721 cells were seeded in 24-well plates at a density of 5 × 10^4^ cells per well and transfected with wild type or mutant luciferase reporter plasmids at the concentration of 50 nmol/L. After 24 hours of incubation, luciferase activity was measured by a dual-luciferase reporter system (Promega, Fitchburg, WI, USA).

### 2.10. Statistical Analysis

Statistical analysis was executed by SPSS 16.0 software and images were obtained with GraphPad Prism 5 Software. The grayscale detection software is Image J. The data are shown as the mean ± standard deviation (SD). The correlation analyses were using Pearson's correlation analyses, and the between-group differences were evaluated using Student's T test or one-way ANOVA. For TMA, the relationship between PHF2 and the clinicopathologic factors of the HCC patients was evaluated by *χ*2 test. The Kaplan-Meier method and log-rank test were employed to assess the correlation between PHF2 expression and patient survival. P<0.05 is identified as statistically significant (*∗*P<0.05, *∗∗P*<0.01, *∗∗∗*P<0.001).

## 3. Results

### 3.1. MiR-221 Expression Is Increased in HCC Cell Lines and Tissues

**Figure 1:**
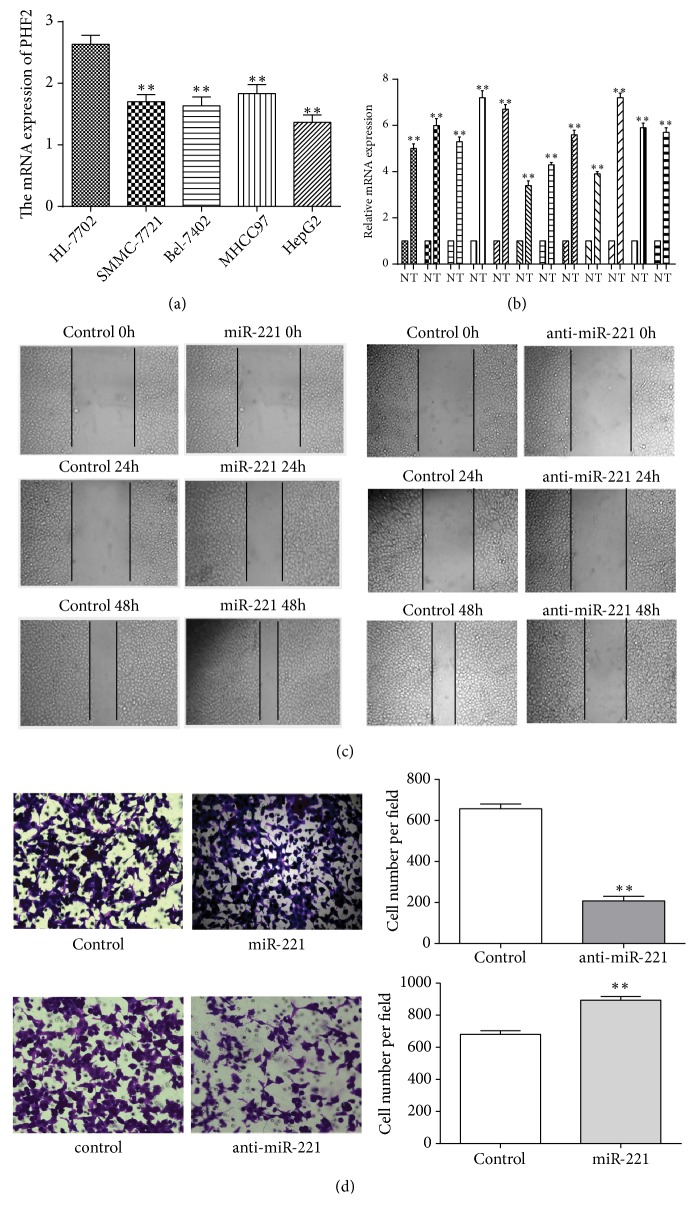
miR-221 is upregulated in HCC tissues and cell lines and promotes the cell migration. (a) qRT-PCR results of miR-221 mRNA levels in HCC cell lines and human normal hepatocyte. (b) Quantitative PCR results of miR-221 mRNA levels in HCC tissues (T) and in adjacent noncancerous tissues (N). MiR-221 mRNA levels are higher in the HCC tissues (T) than in adjacent noncancerous tissues (N) (n=12). (c) miR-221 increases SMMC-7721 cells wound healing. Anti-miR-221 inhibits SMMC-7721 cells wound healing. Lines indicate the border of the healing wounds. (d) The cell migration of SMMC-7721 cells after transfection with miR-221 or anti-miR-221 and their negative control detected by transwell assays. Each bar represents the mean ± SD of three independent experiments. *∗∗P*<0.01.

To investigate the mRNA level of miR-221 in HCC cell lines, qRT-PCR was performed and results demonstrated that the miR-221 mRNA levels in HCC cell lines were higher than human normal hepatocyte (HL-7702) (Figure 1(a)). Moreover, we also found that miR-221 levels were higher in HCC tissues (T) than in adjacent noncancerous tissues (N) (n=36, Figure 1(b), Supplemental Figure 1). Results demonstrated that higher miR-221 expression was evidently associated with HCC. Thus, we used HCC cells to examine the role of miR-221 on cell migration. Furthermore, we demonstrated the correlation of the relative expression of miR-221 with the clinicopathological features of HCC patients in Table 1. Results showed that miR-221 expression in HCC patients with tumor size (≤7cm), pN status (pN_0-1_) or TNM stage II was evidently lower than that with tumor size (>7cm), pN_2_-pN_3_ or TNM stage III-IV (p < 0.05). The relative expression of miR-221 was not found to be associated with age, gender, pT status, or serum AFP levels of HCC patients (p > 0.05). Therefore, miR-221 expression is increased in HCC tissues and had an evident relationship with the HCC patients' characteristics.

### 3.2. MiR-221 Promotes the HCC Cell Lines Migration

To validate whether miR-221 was associated with the migration of HCC cells, we examine the effect of miR-221 on the cell wound healing. We found that miR-221-transfected cells showed a longer distance of shift whereas anti-miR-221-transfected groups showed shorter shift when compared with relevant negative control group (Figure 1(c)). The data showed that miR-221 promoted HCC cells wound healing capability. We then investigated the effect of miR-221 on HCC cell lines migration and found that miR-221-transfected SMMC-7721 cells enhanced the number of cells penetrating the inserts. In contrast, miR-221 inhibition decreased the abilities of SMMC-7721 cells to penetrate the inserts (Figure 1(d)). The results revealed that miR-221 could promote cell migration in SMMC-7721 cells.

### 3.3. MiR-221 Influences Metastasis-Related Genes

**Figure 2:**
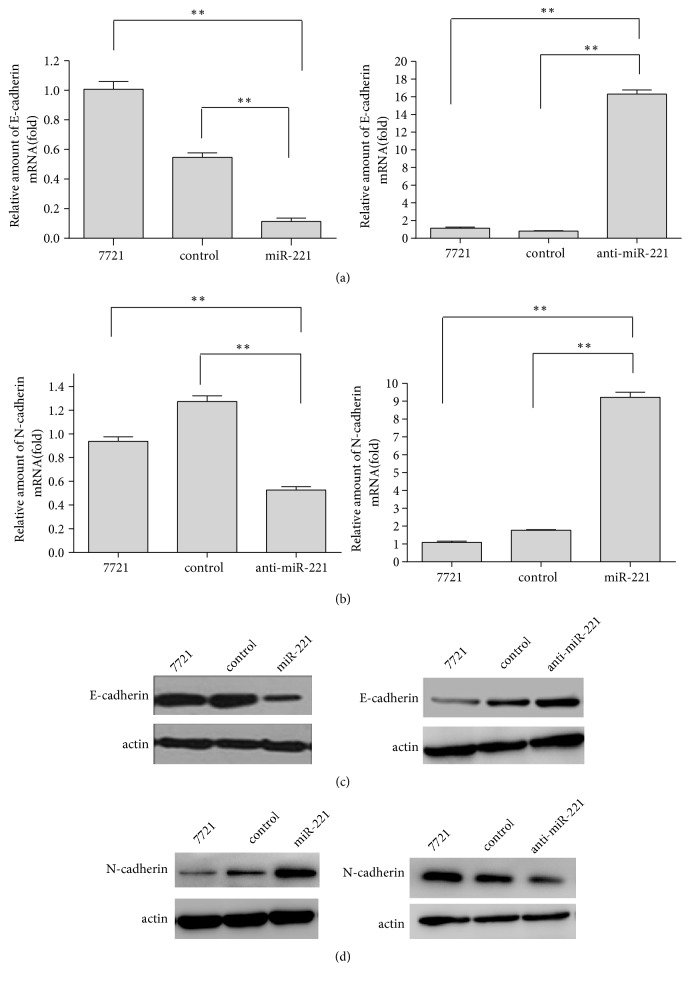
MiR-221 has effects on some key metastasis-related proteins. (a-b) qRT-PCR was used to detect E-cadherin and N-cadherin mRNA level in miR-221-overexpression and miR-221-knockdown SMMC-7721 cells. (c-d) Western blot was used to detect E-cadherin and N-cadherin protein level in miR-221-overexpression and miR-221-knockdown SMMC-7721 cells. Each bar represents the mean ± SD of three independent experiments. *∗∗P*<0.01.

To investigate the effect of miR-221 on cell migration, we carried molecular analyses to detect the expression of some type metastasis-related genes. Our results showed that miR-221 downregulated the expression of an epithelial marker (E-cadherin) in mRNA levels in SMMC-7721 cells, whereas anti-miR-221 led to the opposite results (Figure 2(a)). Meanwhile, the mRNA level of a mesenchymal marker (N-cadherin) was increased when the HCC cells was transfected with miR-221. And anti-miR-221 decreased the expression of N-cadherin in mRNA levels (Figure 2(b)). Furthermore, miR-221 had the same effect on E-cadherin and N-cadherin in protein levels (Figures 2(c) and 2(d)). It is identified that the epithelial-to-mesenchymal transition (EMT) transcription factors play a crucial role in the process of EMT of cancer cells. We performed western blot to verify which EMT transcription factors miR-221 regulates. And the results demonstrated that miR-221 positively regulated the EMT transcription factors Snail and Slug (supplemental Figure 2). These data suggested that miR-221 significantly influenced the expression of EMT transcription factors and biomarkers. Taken together, miR-221 has a crucial impact on HCC cell migration.

### 3.4. PHF2 Is a Target Gene of miR-221

**Figure 3:**
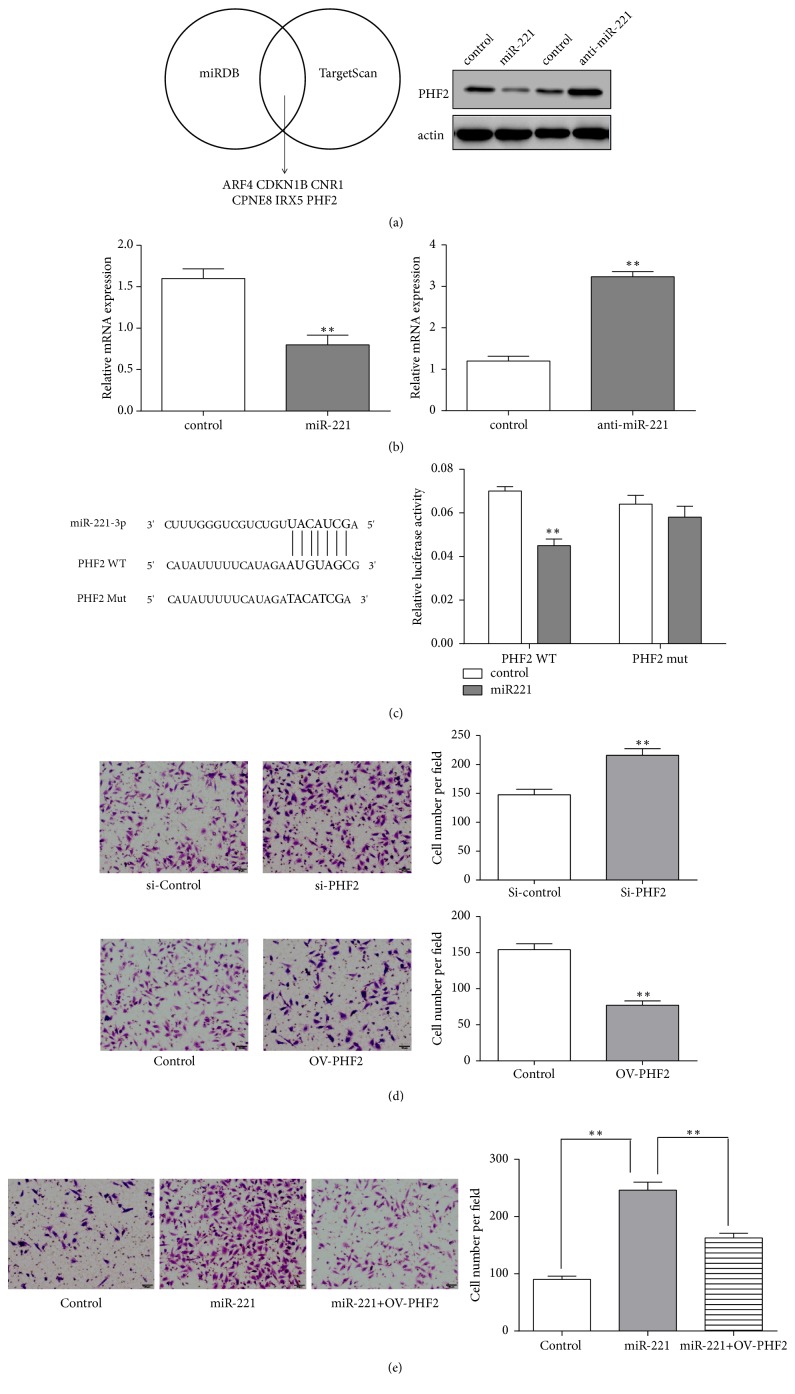
PHF2 is a direct target of miR-221. (a) miRNAs were computationally predicted using two independent miRNA databases. The PHF2 protein level was measured by western blot analysis in SMMC-7721 cells transfected with miR-221 or anti-miR-221. (b) qRT-PCR results of PHF2 mRNA levels after transfecting SMMC-7721 cells with miR-221 or anti-miR-221. (c) Schematic description of wild type (WT) and mutated 3′-UTR of the PHF2 mRNA. The WT and mutated 3′UTR sequences were inserted into luciferase reporter plasmids. In luciferase activity assays, miR-221 suppressed luciferase activity of the wild type but not mutant PHF2 3′-UTR constructs in SMMC-7721 cells. (d) Knockdown of PHF2 promotes the migration of SMMC-7721 cells. Overexpression of PHF2 inhibits the migration of SMMC-7721 cells. (e) Restoration of PHF2 rescues miR-221 mediated promotion of cell migration. Each bar represents the mean ± SD of three independent experiments. *∗∗P*<0.01.

The mechanism of the migration regulated by miR-221 has not been well indicated. Then we found putative genes that miR-221 might regulate by bioinformatics systems. Bioinformatics analysis was performed in two online predicting algorithms miRDB (http://www.mirdb.org/) and TargetScan (http://www.targetscan.org/) to identify miR-221 target genes. Among these genes PHF2 gene was our applicant target. The results showed that miR-221 remarkably reduced the protein PHF2 expression in SMMC-7721 cells. Conversely, anti-miR-221 significantly increased the protein level of PHF2 (Figure 3(a)). In qPCR, our data indicated that miR-221 downregulated PHF2 mRNA levels in SMMC-7721 cells, whereas anti-miR-221 led to the opposite results (Figure 3(b)). To verify whether PHF2 is a target gene of miR-221, we established the dual-luciferase reporter vectors containing mutation type (Mut) or wild type (WT) fragments of PHF2 3′-UTR. The dual-luciferase reporter assay results showed that miR-221 suppressed the activity of luciferase in WT-transfected HCC cells (Figure 3(c)). These results demonstrated the PHF2 3′-UTR is a target of miR-221 in HCC cells.

### 3.5. PHF2 Inhibits HCC Cell Migration

To investigate the role of PHF2 in HCC cell lines, transwell analyses were used to examine the cell migration. The data showed that knockdown of PHF2 increased the number of migratory cells in contrast to negative control group. Meanwhile, following overexpression of PHF2 transfection, the number of migratory cells was decreased compared to negative control group (Figure 3(d)). Thus, PHF2 could inhibit the cell migration of HCC cells. For that PHF2 is a direct target of miR-221 in HCC cells, this is supported by our previous study that miR-221 promoted the migration of HCC cells and anti-miR-221 suppressed the migration of HCC cells in vitro. To investigate the role of miR-221-PHF2 pathway in HCC tumorigenesis, we performed the restoration of PHF2 in miR-221 overexpression cells. Notably, PHF2 rescued miR-221 mediated promotion of migration in SMMC-7721 cells (Figure 3(e)). Collectively, these data indicated that miR-221 could promote cell migration of HCC cells by downregulating PHF2.

### 3.6. The Role of miR-221 and PHF2 Expression in Human HCC

**Figure 4:**
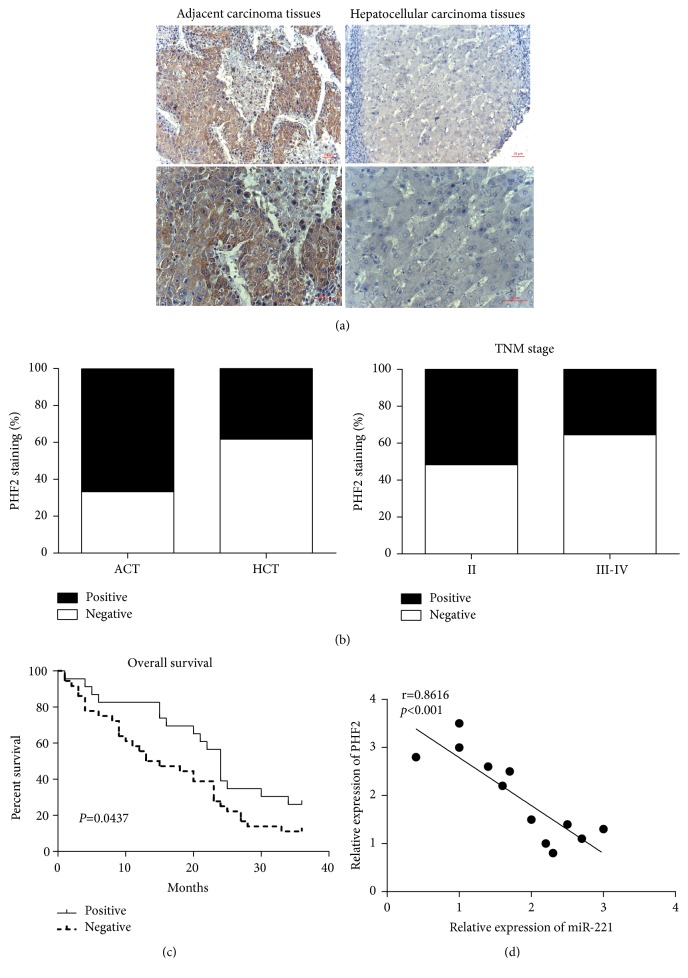
PHF2 is decreased in hepatocellular carcinoma tissues and associated with clinicopathological features in 60 hepatocellular cancer patients. (a) Representative photos of PHF2 expression patterns in hepatocellular tumors. The left panel depicts matched adjacent cancer tissues, whereas the right panel represents the hepatocellular carcinoma tissues (original magnification, ×100). The magnifying detail of the immunohistochemical analysis for each case can be shown in the bottom side (original magnification, ×200). (b) Compared with the adjacent cancer tissues, the overall expression level of PHF2 in the hepatocellular carcinoma tissues was significantly lower, P<0.05, *χ*2 test. PHF2 expression was correlated with TNM stage, P<0.05, *χ*2 test. (c) Kaplan-Meier estimates of the probability of 3-year overall survival according to low and high PHF2 expression of 60 patients with hepatocellular cancer.* P*=0.0437, log-rank test. (d) The correlation between the protein expression of miR-221 and PHF2 was measured by Pearson's correlation coefficient analysis.

We performed immunohistochemistry staining of TMA slide containing HCC/adjacent cancerous tissues and found that PHF2 protein was situated in the cytoplasmic (Figure 4(a)). In adjacent cancerous tissues, high PHF2 staining was recorded in 66.6% (40 of 60 cases). In HCC tissues, high expression of PHF2 was observed in 38.3% (23 of 60 cases). Higher expression of PHF2 was detected in adjacent cancerous tissues compared to the carcinoma tissues (*P*< 0.05, paired *χ*2 test). TNM stage is the most important prognostic indicator for HCC patients, so we investigated whether the protein level of PHF2 was correlated with TNM stage. Our results showed that PHF2 staining was increased in TNM stages II compared with stages III-IV (P < 0.05, paired *χ*2 test) (Figure 4(b)). Furthermore, the protein level of PHF2 was also correlated with lymph node metastasis-pN status, depth of invasion and serum AFP (P < 0.05, paired *χ*2 test) (Table 2). Nevertheless, we did not find the correlation between PHF2 and other clinicopathologic factors including tumor size, age, gender, microvascular invasion, and portal vein tumor thrombus. Overall survival was used for survival analysis and the overall mortality events were 50. The period of the follow-up is 36 months. Kaplan-Meier survival analysis illustrated a higher overall survival in HCC patients with high PHF2 expression than those with low PHF2 expression (P = 0.0437, log-rank test) (Figure 4(c)). To investigate the miR-221 and PHF2 protein levels in vivo, 12 human HCC tissues were detected by the qRT-PCR. The results indicated a negative correlation between the expressions of PHF2 and miR-221 (Figure 4(d)). Furthermore, we performed qRT-PCR and results showed that the PHF2 mRNA levels in HCC cell lines were lower than human normal hepatocyte (HL-7702) (supplemental Figure 4). These suggested that PHF2 was negatively correlated with miR-221 and had an evident relationship with clinicopathological parameters in HCC tissues.

## 4. Discussion

HCC has the highest mortality as a primary cancer for its strong malignant proliferation and migration [13]. The advance diagnosis of HCC largely improves the therapeutic efficacy in patients, thereupon, a sensitive and specific marker is extremely critical [14]. Previous research identified biomarkers mainly focused on proteins [15]; however, miRNAs have absorbed the attention from investigators for the low cost of validation as new molecular markers [16]. Furthermore, dysregulation of miRNAs is a general incident that influences cell invasion, migration, apoptosis, and proliferation in tumor progression [17]. The aim of our study is to investigate the role of miR-221 in HCC and the candidate as diagnostic and therapeutic indicator. Our study demonstrated that the level of miR-221 in HCC is higher than that in adjacent cancerous tissues and cell lines. These indicated that miR-221 could be regarded as a biomarker in early diagnosis and thereby establishing new treatment strategies for HCC.

MiR-221 has been identified to be abnormally regulated in various tumors and involved in cancer cell proliferation and EMT transition in breast cancers [18–21]. Hence, we explored the biological role of miR-221 in HCC cell lines, and our results demonstrate that miR-221 could evidently promote HCC cell migration, while inhibition of miR-221 suppressed the cell migration. It is identified that epithelial-to-mesenchymal transition (EMT) which could induce stem cell features was accompanied by a stable increase in EMT-associated mesenchymal markers and a decrease in epithelial markers [22, 23]. Previous studies have showed that miR-221 increased E-cadherin level in an EMT-induced cell line [23] and miR-221 was downregulated by EMT transcription factor Slug in human breast cancer cells [19]. However, the correlation of miR-221 and N-cadherin in HCC remains to be clarified. In our study, the mRNA and protein level of N-cadherin was increased when the HCC cells were transfected with miR-221. Meanwhile, miR-221 could downregulate the E-cadherin expression in protein and mRNA levels. Although miR-221 could regulate the protein level of E-cadherin, Nassirpour et al. were unable to identify the matching sequence in the 3′UTR [24]. Therefore, E-cadherin could not be a target gene of miR-221. Then we determined the potential target genes of miR-221 with miRDB databases and TargetScan. The genes calculated by algorithms were selected as potential genes of miR-221. PHF2 is found to be most promising among the candidates.

Recently, PHF2 has been shown to act as a tumor repressor associated with p53 in colon and stomach tumor development [12, 25]. Meanwhile, PHF2 are overexpressed in esophageal squamous cell carcinoma (ESCC) and was associated with decreased overall survival of ESCC patients [26]. However, association of PHF2 with the underlying molecular mechanisms in HCC cells is poorly understood. In the current study, our findings showed a negative correlation between PHF2 and miR-221. MiR-221 decreased the expression of PHF2 in both mRNA and protein levels. As shown in luciferase reporter assay, miR-221 inhibited the activity of luciferase in WT-PHF2-3′-UTR transfected HCC cells. These demonstrated that PHF2 3′-UTR was a target of miR-221-3p in HCC cells. We also found PHF2 inhibited HCC cell migration and an evident lower expression of PHF2 was detected in the carcinoma tissues compared with adjacent cancerous tissues. Meanwhile, the protein level of PHF2 was correlated with depth of invasion, TNM stage and lymph node metastasis-pN status. Furthermore, we verified the negative correlation between the mRNA level of miR-221 and PHF2 in clinical HCC patients by Pearson's correlation coefficient analysis. To the best of our knowledge, this is the first study that investigated the role of PHF2 as a target gene of miR-221 in HCC development.

Taken together, this study showed that miR-221 was upregulated in HCC cells and tissues and revealed the tumorigenesis role in HCC cells. Moreover, we also identified PHF2 as a target gene of miR-221 in experimental and clinical levels. Our data indicated that miR-221 participated in HCC cell migration and played its biological roles via regulating the PHF2 gene in HCC. Our characterization of this signaling pathway may provide novel therapeutic targets for the future treatment of HCC.

## Figures and Tables

**Table 1:** Correlation between the HCC patients' clinicopathologic characteristics and miR‐211 expression.

	miR-221 expression		
Variables	n = 36	2^−ΔΔCt^	*P∗*
*Age*			
≤57 years	13	2.63±0.54	0.340
>57 years	23	2.85±0.50	
*Gender*			
Male	26	2.64±0.75	0.415
Female	10	2.48±0.33	
*Tumor size*			
≤7cm	20	2.33±0.37	0.001
>7cm	16	3.31±0.23	
*pT status*			
pT_1-2_	21	2.68±0.52	0.161
pT_3-4_	15	2.98±0.59	
*pN status*			
pN_0-1_	12	2.37±0.40	0.001
pN_2_-pN_3_	24	3.28±0.21	
*TNM stage*			
II	18	2.34±0.37	0.001
III-IV	18	3.28±0.24	
*Serum AFP*			
≤400*μ*g/L	11	2.40±0.47	0.014
>400*μ*g/L	25	2.55±0.47	
*Microvascular invasion*			
Yes	10	2.81±0.62	0.268
No	26	3.09±0.61	
*Portal vein tumor thrombus*			
Yes	9	2.87±0.30	0.323
No	27	3.14±0.43	

**Table 2:** Patients' characteristics and PHF2 expression. *∗*P values are obtained from *χ*2 test.

Variables	PHF2 staining			
	High (%)	Low (%)	Total	*P∗*
*All points*	23 (38.3)	37 (61.7)	60	
*Age*				
≤57 years	5 (33.3)	10 (66.7)	15	0.646
>57 years	18 (40.0)	27 (60.0)	45	
*Gender*				
Male	16 (41.0)	23 (59.0)	39	0.559
Female	7 (33.3)	14 (66.7)	21	
*Tumor size*				
≤7cm	13 (27.9)	25 (72.1)	38	0.388
>7cm	10 (34.5)	12 (65.5)	22	
*pT status*				
pT_1-2_	8 (61.5)	5 (38.5)	13	0.013
pT_3_	12 (46.2)	14 (53.8)	26	
pT_4_	3 (14.3)	18 (85.7)	21	
*pN status*				
pN_0_	10 (58.8)	7 (41.2)	17	0.032
pN_1_	8(44.4)	10 (55.6)	18	
pN_2_-pN_3_	5 (20.0)	20 (80.0)	25	
*TNM stage*				
II	13 (56.5)	10 (43.5)	23	0.022
III-IV	10 (27.0)	27 (73.0)	37	
*Serum AFP*				
≤400*μ*g/L	14(53.8)	12(46.2)	26	0.031
>400*μ*g/L	9(26.5)	25(73.5)	34	
*Microvascular invasion*				
Yes	12(37.5)	20(62.5)	32	0.887
No	11(39.3)	17(60.7)	28	
*Portal vein tumor thrombus*				
Yes	13(41.9)	18(58.1)	31	0.553
No	10(34.5)	19(65.5)	29	

## Data Availability

All relevant data used to support the findings of this study are included within the article.

## Abbreviations

miRNAs: MicroRNAs
PHF2: Plant homeodomain finger 2
HCC: Hepatocellular carcinoma
PHD: Plant homeodomain
TMA: Tissue microarray
HCT: Hepatocellular carcinoma tissues
EMT: Epithelial-to-mesenchymal transition
ESCC: Esophageal squamous cell carcinoma.

## Supplementary Materials

Supplementary Materials Supplemental Figure 1: quantitative PCR results of miR-221 mRNA levels in HCC tissues (T) and in adjacent noncancerous tissues (N). MiR-221 mRNA levels are higher in the HCC tissues (T) than in adjacent noncancerous tissues (N) (n=24). Each bar represents the mean ± SD of three independent experiments. *∗∗*P<0.01. Supplemental Figure 2: western blot was used to detect Snail and Slug proteins level in miR-221-overexpression and miR-221-knockdown SMMC-7721 cells. Supplemental Figure 3: the histograms that express grayscale of Figures 2C-D. Each bar represents the mean ± SD of three independent experiments. *∗∗*P<0.01. Supplemental Figure 4: qRT-PCR results of PHF2 mRNA levels in HCC cell lines and human normal hepatocyte. Each bar represents the mean ± SD of three independent experiments. *∗∗*P<0.01.
