# Supplementary material for: MiR-221 Promotes Hepatocellular Carcinoma Cells Migration via Targeting PHF2
Source: Biomed Res Int. 2019 May 12;2019:4371405. doi: 10.1155/2019/4371405 (PMC6535842; doi:10.1155/2019/4371405)

**Supplemental figure legends:**

**Supplemental figure 1** Quantitative PCR results of miR-221 mRNA levels in HCC tissues (T) and in adjacent non-cancerous tissues (N). MiR-221 mRNA levels are higher in the HCC tissues (T) than in adjacent non-cancerous tissues (N) (n=24). Each bar represents the mean ± SD of three independent experiments. **$P<0.01$

**Supplemental figure 2** Western blot was used to detect Snail and Slug proteins level in miR-221-overexpression and miR-221-knockdown SMMC-7721 cells.

**Supplemental figure 3** The histograms that express grayscale of figure 2C-D. Each bar represents the mean ± SD of three independent experiments. **$P<0.01$

**Supplemental figure 4** qRT-PCR results of PHF2 mRNA levels in HCC cell lines and human normal hepatocyte. Each bar represents the mean ± SD of three independent experiments. **$P<0.01$

## Supplemental figure 1

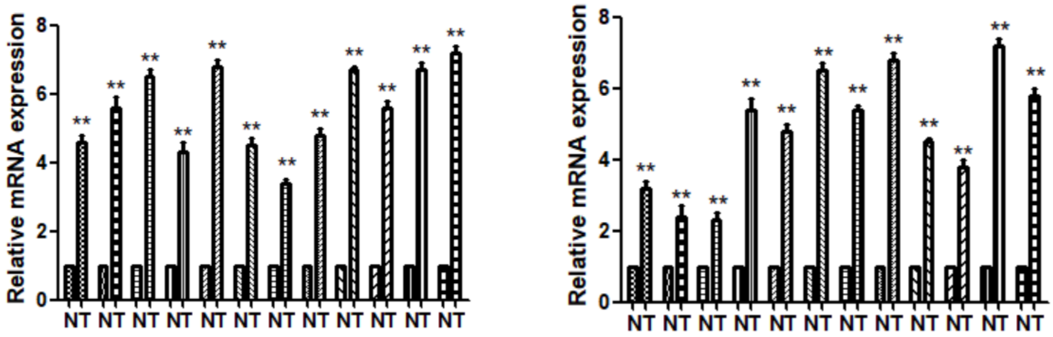


## Supplemental figure 2

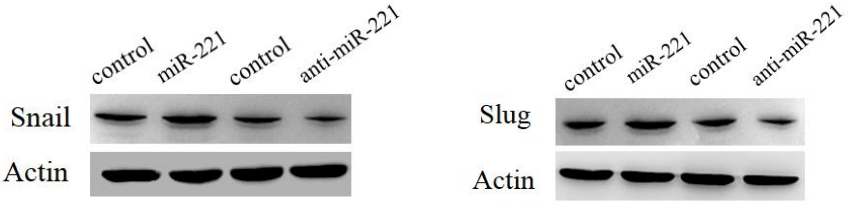


## Supplemental figure 3

## A

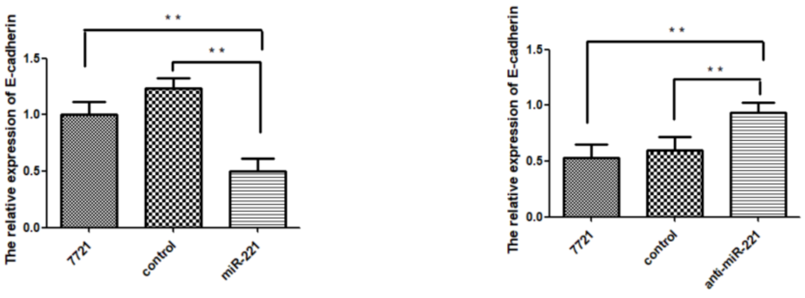


## B

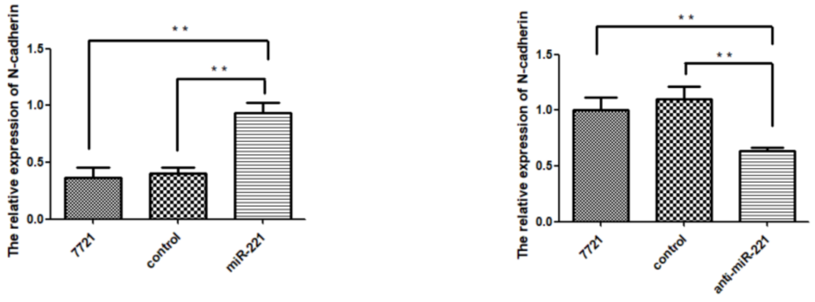

**Supplemental figure 4**

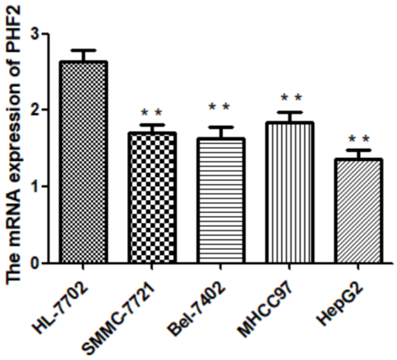

Supplement: Supplementary Materials — Supplemental Figure 1: quantitative PCR results of miR-221 mRNA levels in HCC tissues (T) and in adjacent noncancerous tissues (N). MiR-221 mRNA levels are higher in the HCC tissues (T) than in adjacent noncancerous tissues (N) (n=24). Each bar represents the mean ± SD of three independent experiments. ∗∗P<0.01. Supplemental Figure 2: western blot was used to detect Snail and Slug proteins level in miR-221-overexpression and miR-221-knockdown SMMC-7721 cells. Supplemental Figure 3: the histograms that express grayscale of Figures 2C-D. Each bar represents the mean ± SD of three independent experiments. ∗∗P<0.01. Supplemental Figure 4: qRT-PCR results of PHF2 mRNA levels in HCC cell lines and human normal hepatocyte. Each bar represents the mean ± SD of three independent experiments. ∗∗P<0.01. [file 4371405.f1.zip › source file supplemental material _BMRI_2746138.pdf]
